# Energy offsets between the Er^3+^ 4*f*-states and the GaAs energy levels important for photon ratchet intermediate-band solar cells

**DOI:** 10.1080/14686996.2026.2732505

**Published:** 2026-09-18

**Authors:** Kota Toda, Yuka Kitano, Taiki Kontani, Shigeo Asahi, Yukihiro Harada, Yusuke Oteki, Myeongok Kim, Tatsuki Kawamura, Tomah Sogabe, Yoshitaka Okada, Takashi Kita

**Affiliations:** a Department of Electrical and Electronic Engineering, Graduate School of Engineering, Kobe University, Kobe, Japan; b Research Center for Advanced Science and Technology, The University of Tokyo, Tokyo, Japan; c i-Powered Energy System Research Center, The University of Electro-Communications, Tokyo, Japan

**Keywords:** Photon ratchet intermediate-band solar cells, Er-doped GaAs, oxygen-related state, photoluminescence spectroscopy, density functional theory

## Abstract

The lifetime of the carriers present in the intermediate bands is short, and thus an additional band at slightly lower energies is introduced in photon ratchet intermediate-band solar cells to reduce the impact of the intermediate-band lifetime. This so-called ratchet band should have a long lifetime and allow a fast energy transfer from the intermediate band. Here, we investigate the optical properties of Er-doped GaAs, where the Er atoms provide the ratchet states and the intermediate states are formed by Er-related oxygen defects, to elucidate the actual energy offsets in this system. The temperature dependence of our photoluminescence data shows two activation energies, 214 meV and 10 meV, and we attribute the former to thermal activation from the Er^3+^ excited state labelled ^4^*I*_11/2_ to the GaAs conduction band and the latter to thermal activation from the ratchet state (^4^*I*_13/2_) to localized states related to the oxygen defects. By using photoluminescence excitation spectroscopy, we identified the ^4^*I*_9/2_ state within the GaAs conduction band, which suggests that the Er^3+^ ground-state manifold (^4^*I*_15/2_) is energetically aligned with the GaAs valence-band maximum. These findings are useful in developing strategies that lead to photon-ratchet intermediate-band solar cells with higher efficiencies.

## IMPACT STATEMENT

This study clarifies energy-level alignment and long-lived ratchet states in Er-doped GaAs, enabling rational design of photon-ratchet intermediate-band solar cells toward higher energy conversion efficiency.

## GRAPHICAL ABSTRACT

**Figure:**
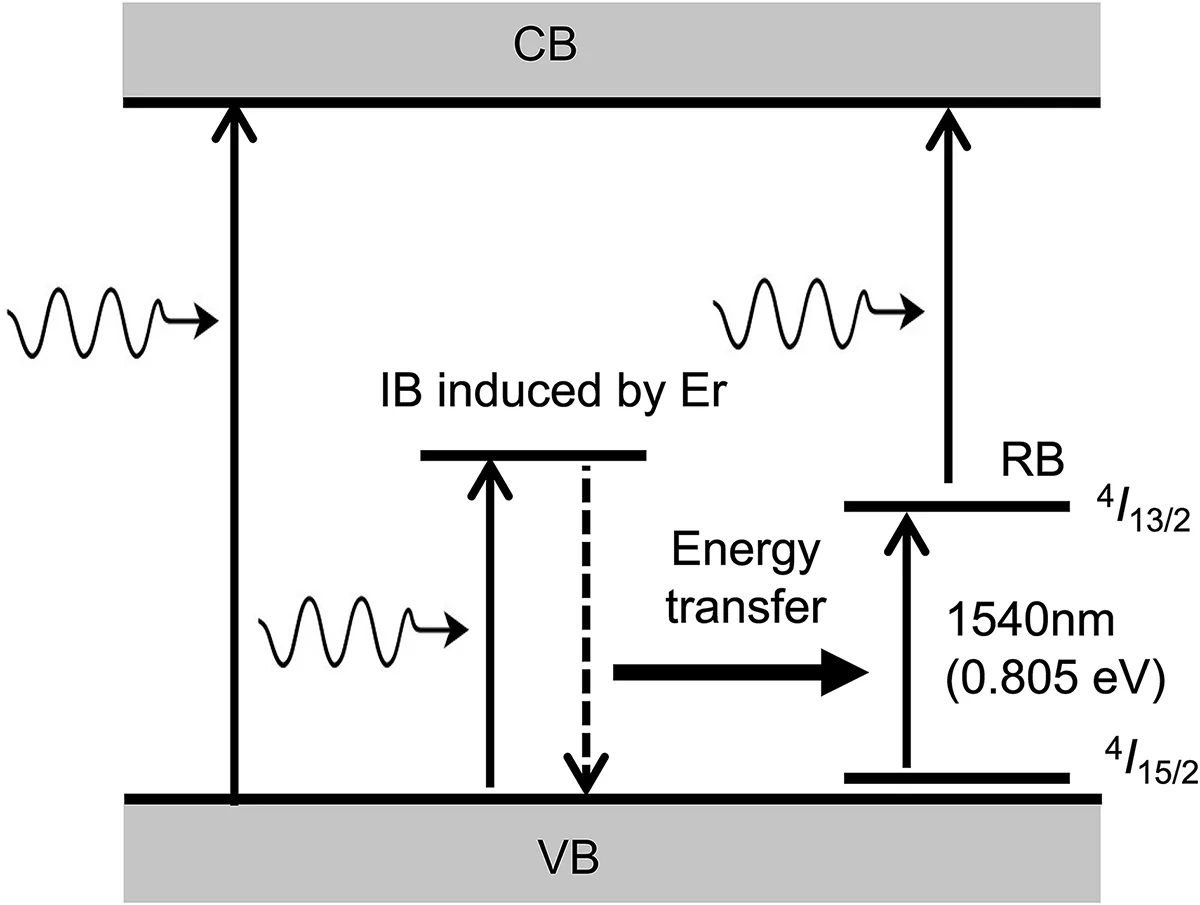

## 1. Introduction

Intermediate-band solar cells (IBSCs) were proposed with the aim of surpassing the energy conversion efficiency of single-junction solar cells, which can only gain high output voltages by significantly narrowing down the range of spectral absorption. To overcome this limitation, the IBSC introduces an intermediate band (IB) between the valence band (VB) and conduction band (CB) in the semiconductor absorber layer [1–4]. This additional band enables a stepwise transition from the VB to the CB via the IB, in contrast to direct VB–CB transitions, and thus, in principle, allows additional absorption of low-energy photons while maintaining the output voltage: Under maximally concentrated solar illumination using the AM1.5D spectrum, the theoretical conversion efficiency of an IBSC can reach 67.6%, if the band configuration is chosen correctly and an idealized photoresponse and carrier transport are considered [3]. In practice, however, the non-ideal electron lifetime in the IB strongly limits the performance [2]. The lifetime in the IB is governed by recombination with the holes in the VB and thermal escape to the CB, and is typically on the order of 10 ns for direct and allowed VB–IB transitions [5,6]. Since a short carrier lifetime in the IB reduces the IB occupancy, the rate of the subsequent IB–CB transition becomes slower, which breaks the current-matching condition and reduces the efficiency. The introduction of a ratchet band (RB) below the IB offers a route to mitigate this limitation [7]: Here, the second step in the carrier generation process by low-energy photons proceeds from the RB to the CB, and if energy stored in the electrons that populate the IB is efficiently transferred to an RB with a long lifetime, the detrimental impact of the relatively short lifetime in the IB can be suppressed.

In the case of optically forbidden RB–VB transitions, the carrier lifetime in the RB is limited only by thermal escape to the IB and the CB, and rare-earth ions are known to be promising candidate materials for such RBs [8]. The intra-4*f* transitions in these ions are forbidden, resulting in excited-state lifetimes on the order of milliseconds [9–11]. A photon ratchet IBSC based on Er-doped GaAs (GaAs:Er) has recently been proposed, and preliminary data of an actually fabricated device has been discussed [8]. Since the lifetime of the carriers present in the RB depends on the separation between the RB and IB energy levels, the design of an IBSC with an RB requires a precise knowledge of various energy levels. In this work, we investigate the properties of GaAs:Er thin film grown on a GaAs (001) substrate by photoluminescence (PL) spectroscopy and density functional theory (DFT) to obtain more knowledge about the actual energy offsets in this system.

## 2. Methods

### 2.1. Sample preparation

The GaAs:Er thin film investigated in this study was grown on a semi-insulating GaAs (001) substrate by molecular beam epitaxy. After surface-oxide desorption of the GaAs substrate, a 250-nm-thick intrinsic GaAs buffer layer was grown at a substrate temperature of 580°C. Then, a 250-nm-thick GaAs:Er layer with an Er concentration of 1.5 × 10^18^ cm^−3^ was grown at a growth rate of 1 μm/h, where Er was supplied simultaneously with Ga under an As_2_ flux from a cracker cell with a beam equivalent pressure of 2.0 × 10^−5^ Torr. The Er cell temperature was 800°C. Finally, an intrinsic GaAs capping layer was deposited. The Er concentration was evaluated using a secondary ion mass spectrometer (CAMECA IMS-7f, O_2_^+^ at 3 keV, raster size: 30 μm^2^).

### 2.2. PL measurements

The PL data were recorded in vacuum at various temperatures using a cryostat with a temperature controller. The excitation power dependence and the temperature dependence of the PL were measured using the same system, and the PL excitation (PLE) measurement was performed using a different system. For the excitation power dependence, we used 808-nm light from an infrared laser diode with an excitation intensity between 5.1 MW/cm^2^ and 15 W/cm^2^ varied using optical density filters and a beam spot with a diameter of 0.5 mm. The emitted PL signal was collected through an optical fiber, dispersed by a monochromator (Acton Research Corporation, USA, SP-2300i) equipped with a grating with 150 gr/mm and a blaze wavelength of 1200 nm, and detected by an InGaAs diode array (Princeton Instruments, USA, 7498–0001) cooled to approximately −90°C. The incident power was measured directly in front of the sample using an optical power meter, and the sample temperature was 7.5 K. The temperature-dependent PL measurements were conducted in the temperature range 7–280 K, while the excitation intensity was fixed to 5.1 W/cm^2^. The PLE data were recorded at 3 K using monochromatic light from a supercontinuum white-light laser (NKT Photonics A/S, Denmark, EXR-15) and a monochromator (SPEX Industries, Inc., USA, 270 M) equipped with a grating with 300 gr/mm and a blaze wavelength of 2000 nm. The excitation wavelength was scanned over the range from 780 nm to 1000 nm in steps of 2 nm. The spectral linewidth of the excitation light was approximately 3 nm in terms of the full width at half maximum (FWHM) obtained from fitting using a Lorentzian line shape. The emitted PL was collected through an optical fiber and detected by a near-infrared spectrometer (Ocean Optics, Inc., USA, NIRQ512).

### 2.3. DFT calculations

An Er-doped GaAs supercell with zinc-blende structure (4 × 4 × 4 unit cells) was constructed by replacing one Ga atom with Er, yielding Ga_255_As_256_Er_1_, and the required data for Er was obtained from the Materials Project database [12,13]. To examine the oxygen-related effects that have been frequently reported in experimental studies of GaAs:Er – the incorporation of oxygen is unavoidable during Er doping and the formation of Er–O complexes is closely associated with efficient Er-related luminescence centers – we also considered a supercell in which the effect of oxygen codoping is introduced by replacing two As atoms with O, yielding Ga_255_As_254_Er_1_O_2_ [14–17]. Geometry optimization was performed using the software QuantumATK (version R-2020.09-SP1) [18,19] with the GGA-PBE exchange–correlation functional, FHI pseudopotentials, and a double-zeta polarized basis. We considered electronic smearing corresponding to 300 K and used a density mesh cut-off energy of 45 Hartree, and the band structure and the projected densities of states (DOS) were computed for both Ga_255_As_256_Er_1_ and Ga_255_As_254_Er_1_O_2_. To analyze the electronic structure of the relaxed geometry with an improved treatment of localized rare-earth states, hybrid-functional calculations were further carried out with the HSE06 functional using the same pseudopotentials and the same basis. This approach is motivated by (i) the known difficulty in describing Er 4f electrons by semi-local DFT and (ii) prior work that reports an improved agreement with experimental structural and electronic properties in rare-earth compounds if the HSE06 functional is used [20,21]. While a more accurate estimation of the band-gap can be expected with this method, an uncertainty with respect to the absolute positions of the states within the gap remains.

## 3. Results and discussion

### 3.1. The lifetime of the ^4^I_13/2_ state in GaAs

Figure 1(a) shows the PL spectra of the GaAs:Er sample with unintentional oxygen codoping for excitation power densities ranging from 5.1 MW/cm^2^ to 15 W/cm^2^ at 7.5 K. A PL peak is observed at 1540 nm, which corresponds to the transition from the lowest excited state of Er^3+^, ^4^*I*_13/2_, to the ground state, ^4^*I*_15/2_. The spectral shape of this peak is almost independent of the excitation power density. On the low-energy side of this peak, above 1540 nm, several emission lines with an intensity-ratio similar to the emission reported for Er centers, and different types of Er centers can form depending on the growth temperature [14]. The PL of the so-called Er–2O centers can also appear above the main peak at 1538 nm [22]. It is known that oxygen atoms incorporated during growth tend to form Er–2O centers, which cause crystal field splitting as well as spin-orbit splitting of the ^4^*I*_13/2_ and ^4^*I*_15/2_ levels [14,15,23–26]. The split energy levels appear as the low-energy shoulder of the main peak in Figure 1(a).

**Figure 1.**
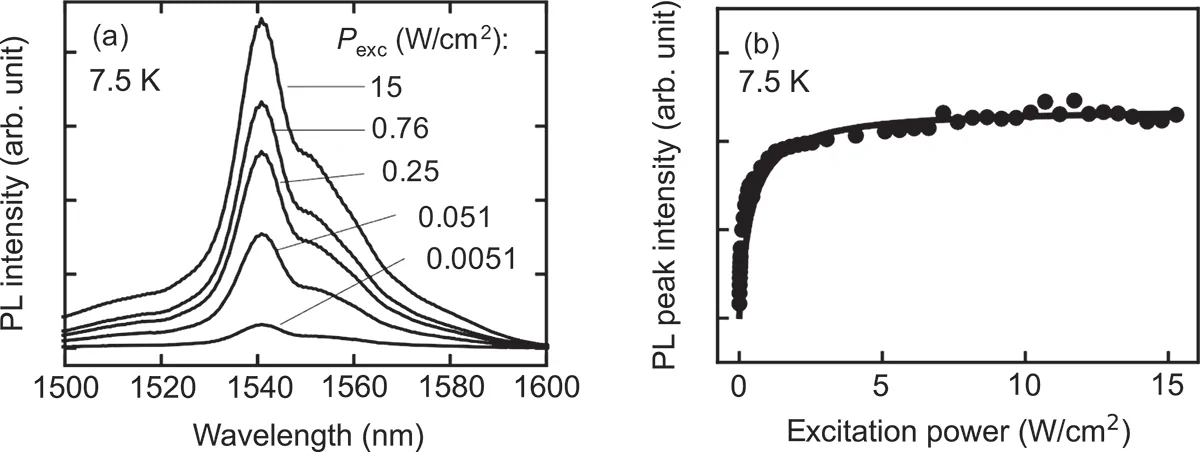
(a) The PL spectra of GaAs:Er at 7.5 K for various excitation power densities. (b) The excitation power dependence of the amplitude of the PL peak at 1540 nm. The solid line indicates the fitted result using Equation (1).

Figure 1(b) shows the excitation power dependence of the amplitude of the main peak. A saturation of the PL intensity can be confirmed, and we performed curve fitting using a model that considers the excitation cross-section *σ* and the lifetime *τ* of the lowest excited state [27–29]:

(1) $$\begin{array}{c} I_{\mathrm{PL}}\propto \frac{\sigma \tau F_{\mathrm{ex}}}{\sigma \tau F_{\mathrm{ex}}+1} \end{array}$$

where *F*_ex_ denotes the excitation photon flux density. In this experiment, the Er^3+^ ions are indirectly excited by the incident light at 808 nm via an energy transfer to the 4*f* states after optical absorption in the GaAs host. Therefore, *F*_ex_ is the photon flux that excites the GaAs host and the cross-section for energy transfer from the GaAs bulk region to the Er ions depends on the Er density. Estimating the radiative lifetime of rare-earth ions from the saturation behavior of the emission intensity, after accounting for the excitation cross section, is theoretically sound and, in principle, feasible. The method relies on the steady state rate equations of a simple two level system under continuous wave excitation. If excitation cross section *σ* is known, the radiative lifetime follows directly [27–29]. On the other hand, the model is simple, and real rare earth doped materials often deviate from ideal two level behavior, introducing significant uncertainty in the extracted lifetime. For example, high excitation intensities required to reach saturation lead to large excited state populations. Under these conditions, processes such as the depopulation of the ^4^*I*_13/2_ state by optically induced RB–CB transitions, energy transfer up-conversion and excited state absorption become prominent. These introduce nonlinear loss terms into the rate equations, making it difficult to disentangle saturation caused by finite lifetime from saturation caused by up-conversion losses. As a result, the inferred *τ* is typically underestimated. On top of that, intense excitation can induce heating through nonradiative relaxation. Elevated temperatures enhance phonon assisted nonradiative decay, effectively shortening the observed lifetime. Moreover, these excitation intensity dependent uncertainties in the extracted lifetime are affected by excitation beam spatial profile. When direct time-resolved measurements are not accessible, or when complementary insight into excited-state dynamics is desired, this approach can still provide valuable information.

The obtained fitting result is shown by the solid curve, which corresponds to a value of 1.18 × 10^−18^ s⋅m^2^ for the product *στ*. Based on the excitation cross-section of the ^4^*I*_13/2_ state in GaAs:Er reported in the literature (*σ* = 1 × 10^−15^ cm^2^) [29], we can thus estimate an excited state lifetime of 1 × 10^−3^ s in our sample. This estimated lifetime is consistent with previously reported millisecond-scale lifetimes of the ^4^*I*_13/2_ state in Er-doped GaAs and related III–V semiconductor systems determined using time-resolved PL measurements. Furthermore, this long lifetime suggests that an electronic configuration with a high potential energy, i.e. the electrons stored in the excited state, can be maintained for a sufficiently long time to create a large carrier density in the ^4^*I*_13/2_ level without significant recombination losses [2].

Long carrier lifetimes are of particular importance in photon ratchet IBSCs, where carrier storage within the RB is one prerequisite for maintaining a large quasi-Fermi level splitting. The millisecond-scale lifetime observed in GaAs:Er suggests that the Er^3+^ 4*f* excited states, in particular the ^4^*I*_13/2_ manifold, can sustain a large population of excited carriers for sufficiently long times. In this regard, these results suggest that the ^4^*I*_13/2_ state satisfies a key requirement for the photon-ratchet mechanism in an IBSC.

### 3.2. Energy offsets between the Er^3+^ 4f-states and the CB, VB and RB states

To elucidate the relative positions of the energy levels of the Er^3+^ 4*f*-states with respect to the GaAs band structure and the RB, we investigated the temperature dependence of the PL and the shape of the PLE spectrum. Before we consider this data, we briefly discuss the expected trend: Since the thermal energy at about 7 K (0.6 meV) is significantly smaller than the energy separations of the split ^4^*I*_15/2_ states, almost all electrons populate the lowest sublevel [30] Consequently, the optical transitions originating from this state constitute the dominant PL contribution. In relation to this peak, the PL sideband should become stronger with temperature, while the overall PL intensity should decrease. Note that the position of this peak is almost independent of the temperature [8]. We confirmed that this PL peak exhibits only a slight redshift of 2.1 nm as the temperature is increased from 7 K to 180 K, which corresponds to a temperature coefficient of ~0.012 nm/K (this data is not shown here). This trend can be explained with the fact that the outer 5*s* and 5*p* electron shells shield the intra-4*f* transitions of Er^3+^ and thus reduce the impact of lattice expansion and electron–phonon interactions [31].

Figure 2 shows the temperature dependence of the amplitude of the main PL peak as an Arrhenius plot. The PL-intensity quenching is relatively strong and suggests the presence of an energy back-transfer process from the emission center to higher-lying states. The observed temperature response was fitted using the Arrhenius law for non-radiative recombination with two components:

(2) $$\begin{array}{c} I_{\mathrm{PL}}\propto \frac{1}{1+A_{1}\exp\left(-E_{a1}/k_{B}T\right)+A_{2}\exp\left(-E_{a2}/k_{B}T\right)} \end{array}$$

**Figure 2.**
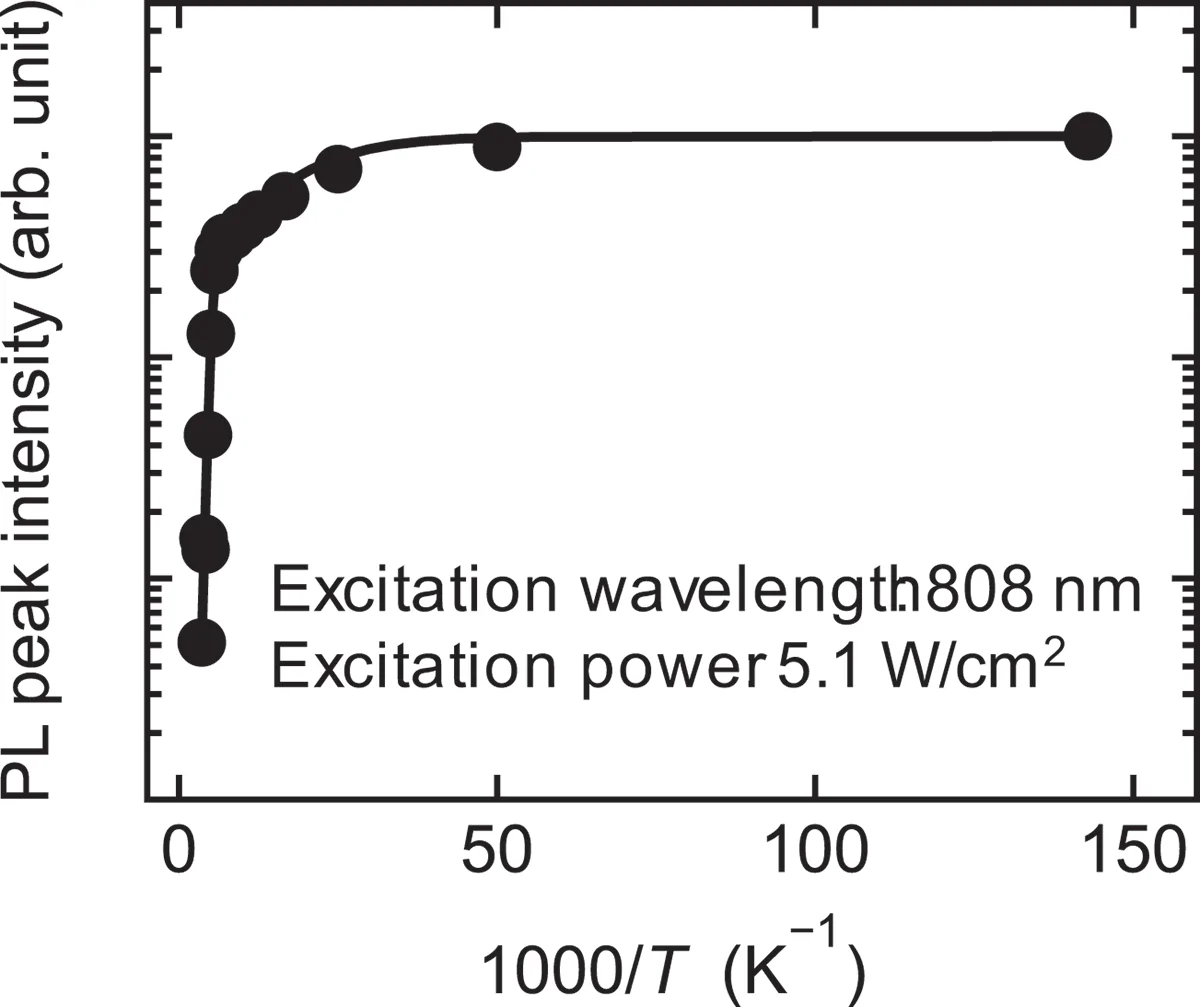
The temperature dependence of the amplitude of the main PL peak for an excitation power density of 5.1 W/cm^2^. The solid line indicates the fitted result using Equation (2).

where *k*_B_ is the Boltzmann constant, *T* is the temperature, *E*_a1_ and *E*_a2_ represent the barrier heights for thermal activation from the lowest excited state to two other states at higher energies, and *A*_1_ and *A*_2_ are the so-called frequency factors, which are required to correctly weight the three considered recombination paths. Here, a scaling factor was additionally introduced during the fitting procedure because the measured PL intensity was not normalized to an absolute emission efficiency. The fitting parameters were the scaling factor, *A*_1_, *A*_2_, *E*_a1_, and *E*_a2_. A model with a single activation energy could not adequately reproduce the experimental temperature dependence, whereas the two-component model shown in Equation (2) yielded a satisfactory fit. The obtained fitting curve is shown by the solid curve in Figure 2, and the obtained fitting parameters are *E*_a1_ = 10 ± 3 meV, *A*_1_ = 4.59 ± 1.84, *E*_a2_ = 214 ± 21 meV, and *A*_2_ = 1.33 × 10^6^ ± 1.32 × 10^6^. The decrease in the PL intensity can thus be separated into two temperature regions: A weak reduction above ~80 K due to activation to an energy level 10 meV above the lowest excited state, and a strong quenching above ~180 K, where activation to an energy level 214 meV above the lowest excited state becomes dominant. The provided threshold temperatures correspond to the condition $e^{-E_{ai}/\left(k_{B}T\right)}$  ≈ 1, which roughly describes the onset of the thermally activated process. The large value of *A*_2_ means that the corresponding process provides an efficient excitation pathway above 180 K due to thermal excitation to a state that can be occupied by many carriers in a bulk state, whereas the small value of *A*_1_ indicates an ineffective quenching process above 80 K due to rapid repopulation through reverse transitions due to thermal excitation to a state with a strongly restricted number of carriers in a localized state [32]. Hence, by evaluating the activation energies, the energy positions of the 4*f*-states within the GaAs bandgap can be inferred.

To clarify the details of the excitation pathways and the energy-level offsets, we measured the PLE spectrum at 3 K by monitoring the amplitude of the PL peak at 1540 nm: In Figure 3, we plotted the PL signal divided by the used excitation photon density, which varied by a factor of ~3.5 throughout the measured wavelength range. The typical excitation density was 21 mW/cm^2^, and at such the weak excitation densities, the PL intensity is linearly proportional to the excitation density. The inset shows the monitored region for an excitation wavelength of 824 nm. Figure 3 clarifies that the PLE spectrum basically follows the absorption-edge profile of GaAs [33], which indicates that the excitation mechanism of the Er^3+^ ions is governed by an energy transfer after the optical absorption in the GaAs host and not by direct excitation. The red dashed line shows the optical absorption profile of undoped GaAs at 12 K obtained from photocurrent measurements [34]. In contrast to the step‑like absorption edge of undoped GaAs, the PLE spectrum of GaAs:Er exhibits a pronounced peak near the GaAs band edge at 817 nm (1.519 eV). The reported energy splitting between the ^4^*I*_9/2_ and ^4^*I*_15/2_ states is 810 nm (1.531 eV), which lies very close to the GaAs band edge. This enhanced absorption suggests the presence of resonant energy transfer from the GaAs CB to the ^4^*I*_9/2_ state, which enables efficient energy relaxation to the ^4^*I*_13/2_ state: When photons with an energy near the GaAs band-edge are strongly absorbed, they generate hot carriers and excitons, which can subsequently transfer their energy to the Er^3+^ ions [35]. If we assume that the 4*f* energy separations are almost identical to the values reported in the literature [36], the observed resonance energy suggests that the ^4^*I*_15/2_ ground state is located near the GaAs VB edge. Furthermore, a weak but still significant PLE intensity is detected on the low-energy side of the GaAs absorption edge in the wavelength range 830–1000 nm. Such a behavior can be attributed to localized electronic states introduced by Er doping.

**Figure 3.**
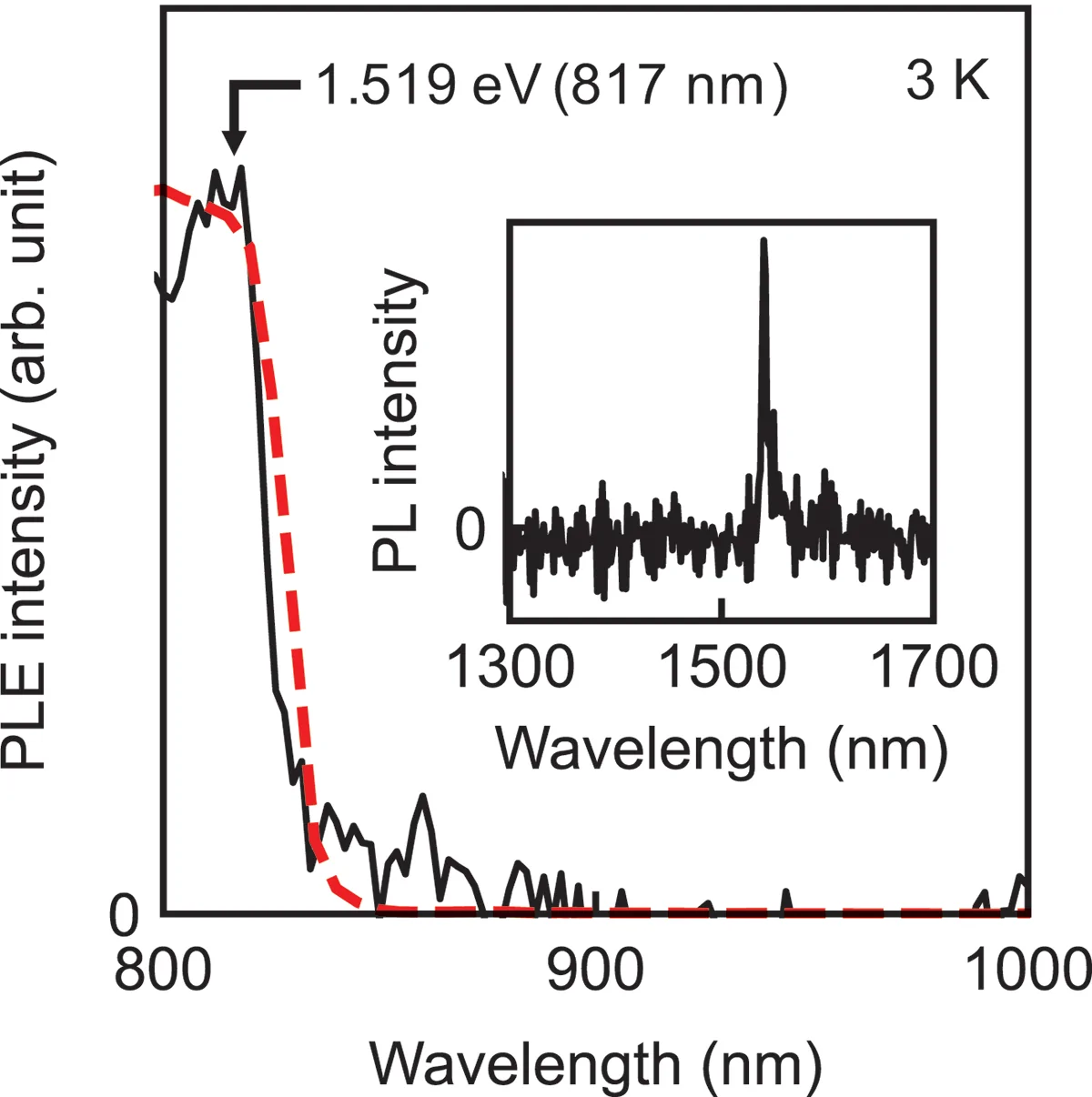
The PLE spectrum of GaAs:Er at 3 K monitored at 1540 nm. The inset shows the PL spectrum of the sample under 824-nm excitation. The red dashed line shows the optical absorption profile of undoped GaAs at 12 K obtained from photocurrent measurements [34].

To support the above interpretation, first-principles calculations were performed, and in Figure 4 we compare the room-temperature properties GaAs:Er and oxygen-codoped GaAs:Er with the same Er concentration (0.39%): Figures 4(a,b) show the band structures and projected DOS for the supercell structures Ga_255_As_256_Er_1_ and Ga_255_As_254_Er_1_O_2_, respectively. It is well known that oxygen is commonly incorporated during Er doping and that Er–O complexes are closely linked to efficient Er-related luminescence centers and oxygen-assisted excitation behavior in GaAs [14,15,20,21]. Figure 4(a) reveals that the GaAs host-band dispersion remains largely preserved in the Ga_255_As_256_Er_1_ supercell, with valence states dominated by Ga/As s–p character and a strong As 4p contribution near the VB edge, indicating that dilute Er substitution does not lead to a strong reconstruction of the host-band framework. The Er projected DOS shown on the right-hand side exhibits a sharp, localized feature associated with the Er 4f manifold, confirming the atomic-like nature of the Er-derived states just above the VB maximum, but no well-isolated level is identified within the GaAs bandgap. In contrast, Figure 4(b) for a supercell with additional O atoms at As sites shows nearly dispersionless bands inside the GaAs gap at about 0.48, 0.55, and 0.78 eV. The O projected DOS shown on the right-hand side displays pronounced contributions to these features, indicating that the well-isolated levels within the GaAs bandgap are oxygen-related (primarily due to O 2p) rather than due to a simple shift of the host bands. Furthermore, the projected DOS suggests coupling to the Er-centered local environment, consistent with the considered presence of an Er–O-associated complex [see the oxygen-projected DOS and the corresponding Er-related peaks at these energies in Figure 4(b)]. Hence, this comparison in Figure 4 demonstrates that oxygen incorporation can generate genuine intermediate electronic levels in dilute GaAs:Er, providing a microscopic basis for introducing oxygen-related IB states in the model used for interpreting our measurement results.

**Figure 4.**
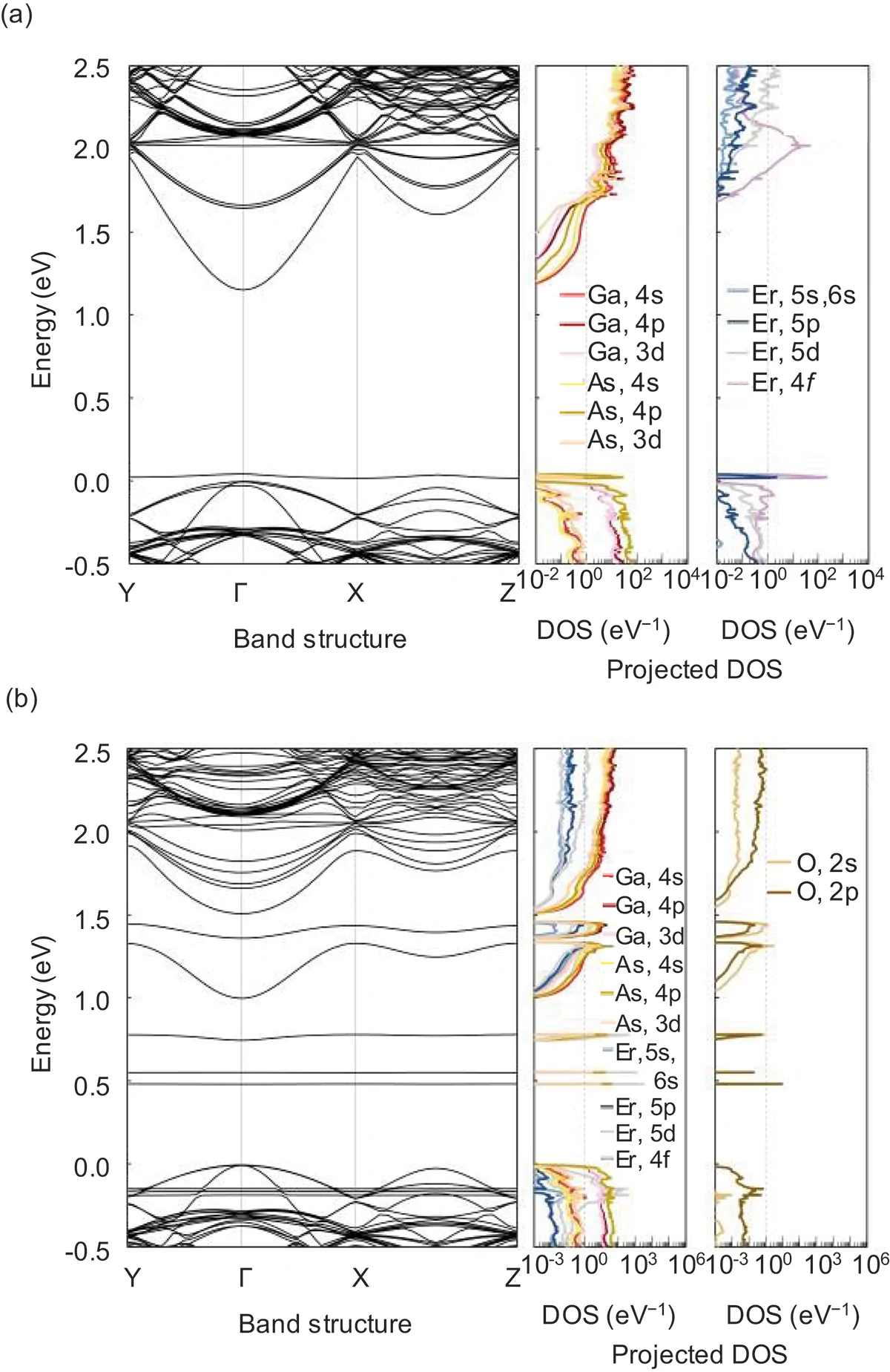
(a) The band structure and projected DOS of dilute Er-doped GaAs (supercell structure: Ga_255_As_256_Er_1_, which corresponds to an Er content of 0.39%) and (b) that of Er-doped GaAs with oxygen codoping (supercell structure: Ga_255_As_254_Er_1_O_2_). Both data sets were calculated using the HSE06 hybrid functional and the band structure is shown along the line that connects the high-symmetry points Y, γ, X, and Z in the first Brillouin zone. For panel (a), the Er-projected DOS is shown separately to highlight the Er contribution, and for panel (b), where two as atoms are replaced by O, the O-projected DOS is shown separately.

Based on the temperature dependence of the PL and the PLE results, as well as the theoretical predictions, the proposed energy-level diagram of GaAs:Er relevant for photon ratchet IBSCs is summarized in Figure 5. The energy separations of the Er^3+^ 4*f*-states were taken from the literature [36]. We did not determine the energy spacings of these states using DFT, because of an uncertainty with respect to the absolute positions of the states within the gap. Therefore, the ^4^*I*_9/2_ state does not appear in Figure 4(a), but the PLE resonance at 1.533 eV in Figure 3 indicates that the ^4^*I*_9/2_ state of Er^3+^ is located in the CB. According to the reported energy separation between the ^4^*I*_9/2_ and ^4^*I*_15/2_ states, the ^4^*I*_15/2_ level should be located near the VB maximum, and this result is consistent with the theoretical prediction shown in Figure 4, in which the mixing between the VB states and the ^4^*I*_15/2_ state is significant. Because the 4*f* energy levels of Er^3+^ are insensitive to the temperature, the PL temperature dependence predominantly reflects changes in the energy-level offsets between the Er^3+^ states and the GaAs host. Therefore, the activation energies extracted from Figure 2 provide information about these changes: The relatively large activation energy *E*_a2_ is interpreted as the offset between the ^4^*I*_11/2_ level and the GaAs CB minimum, which results in an efficient energy back-transfer at higher temperatures. Furthermore, thermally activated quenching processes in rare-earth-doped III–V semiconductors have also been discussed in terms of an energy back-transfer process mediated by host-related carrier trap-states. For example, Taguchi and Takahei proposed a multi-phonon-assisted energy transfer model, in which excitation and de-excitation of the Er^3+^ 4*f* shell occur via recombination and generation of electron–hole pairs at rare-earth-related trap levels in the host semiconductor, respectively [37]. As shown above, introducing O atoms on As sites should produce several nearly dispersionless levels inside the GaAs gap, and one of these additional energy levels with a relatively low DOS appears in the vicinity of the energy level of the ^4^*I*_13/2_ state. Therefore, the relatively small activation energy *E*_a1_ should represent the energy offset between these states, which needs to be overcome to induce the weak thermal quenching of the 4*f*-luminescence observed in Figure 2. Although the quenching mechanism cannot be uniquely identified, the activation energies expected from the energy back-transfer model and the carrier-transfer model become approximately identical, because the energy offset between the ^4^*I*_15/2_ manifold and the VB maximum is expected to be small. Therefore, within the accuracy of the present experiments, both models lead to nearly the same estimate of the energy separation between the defect-related state and the ^4^*I*_13/2_ manifold. Consequently, the interpretation of the energy level alignment shown in Figure 5 is not expected to depend strongly on the specific quenching mechanism. Note that the calculation of the *absolute* positions of the states within the gap with respect to both the VB and the CB is difficult, but the confirmed energy offset *E*_a1_ is consistent with the rather small magnitude of the energy offsets between the localized Er–2O levels and the ^4^*I*_13/2_ level that can be expected based on the energy spacings with respect to the ground state.

**Figure 5.**
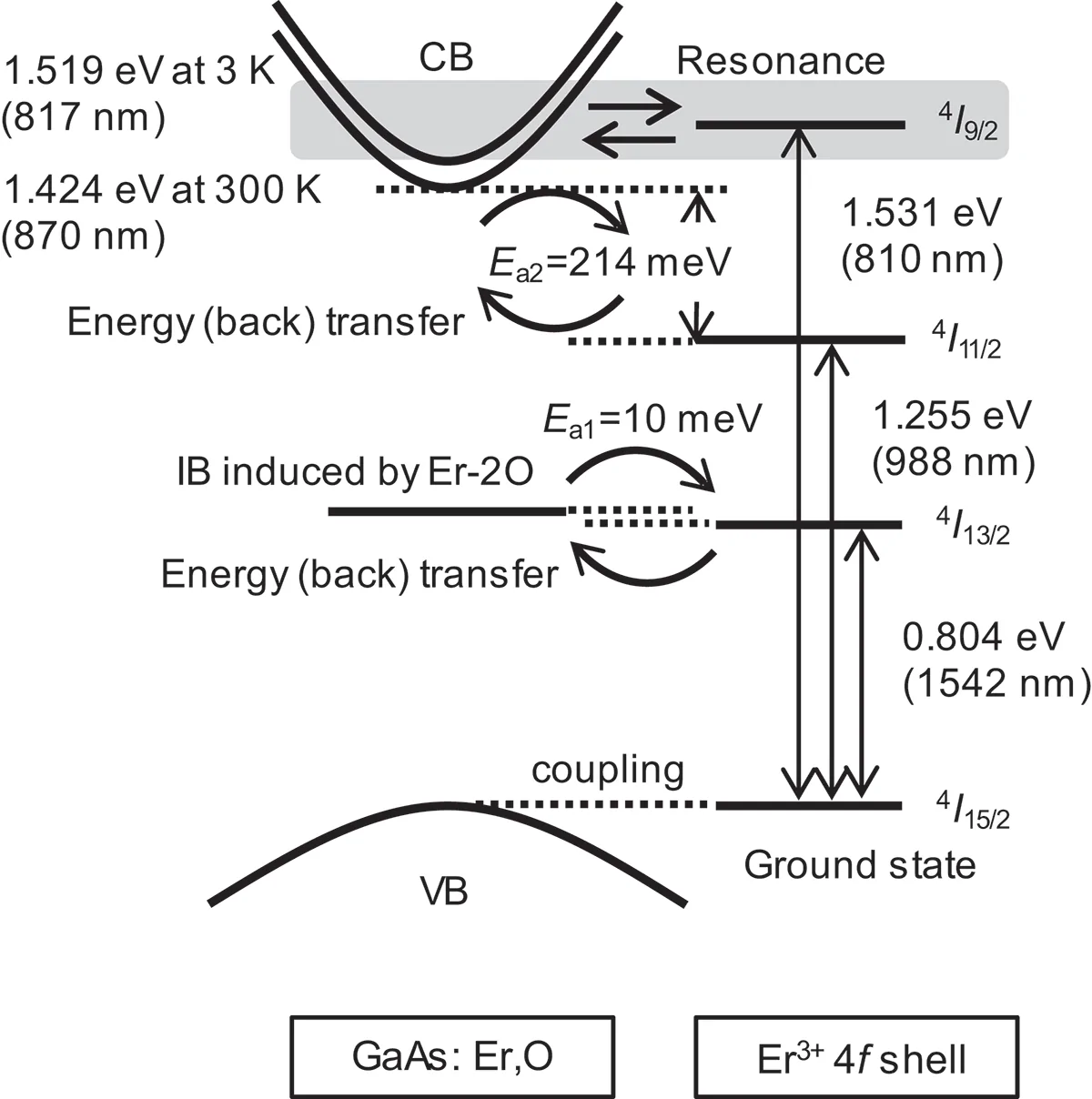
Energy offsets between the Er 4*f*-states and the GaAs CB and VB, as well as the oxygen-defect level for the IB based on the temperature dependence of the PL and the PLE measurements.

## 4. Conclusions

By analyzing the optical properties of GaAs:Er grown on GaAs (001), we have elucidated the energy offsets between the Er^3+^ 4*f* states and the GaAs CB and VB, as well as the roles of oxygen‑induced defect states. The PL intensity of the ^4^*I*_13/2_ state saturated as the excitation power was increased, and fitting with a standard saturation model yielded a product of 1.18 × 10^−18^ s⋅m^2^ for *στ*, corresponding to a lifetime of approximately 1 × 10^−3^ s. This long lifetime implies that the ^4^*I*_13/2_ state can fulfill the following requirement for an RB: the suppression of backward IB–VB transitions by storing the carriers that reached the IB for more efficient sequential two-photon absorption. The temperature dependence of the PL intensity revealed two thermally activated quenching processes: The higher activation energy was attributed to an energy back-transfer process from the ^4^*I*_11/2_ level to the GaAs CB minimum, and the lower activation energy was interpreted in terms of a small energy gap between the localized Er–2O levels and the ^4^*I*_13/2_ level. Therefore, the Er–2O states can be used as an IB of a photon ratchet IBSC, although the energy separation is not enough for operation at room temperature. The PLE spectrum basically follows the GaAs absorption-edge profile, indicating that the excitation of Er^3+^ proceeds predominantly through host absorption rather than direct excitation of the 4*f*-states. In addition, the pronounced enhancement observed around 809 nm in the PLE spectrum demonstrates that the ^4^*I*_9/2_ state is located within the GaAs CB, which suggests that the Er^3+^ ground-state manifold (^4^*I*_15/2_) is energetically aligned with the GaAs VB maximum. From DFT calculations for GaAs:Er (Er concentration of 0.39%) with and without oxygen codoping, we found that oxygen incorporation leads to electronic states inside the GaAs band gap that are absent in the model without oxygen-induced defects, indicating that oxygen codoping can generate intermediate electronic states in GaAs:Er. These findings shed more light on the carrier-generation processes in a photon ratchet IBSC based on long-lived 4*f-*states of Er^3+^ and localized Er–2O states, which act as the RB and IB, respectively.

## Data Availability

The data that support the findings of this study are available from the corresponding author, T.K., upon reasonable request.

## References

[1] Luque A, Martí A. Increasing the efficiency of ideal solar cells by photon induced transitions at intermediate levels. Phys Rev Lett. 1997;78(26):5014–9. doi: 10.1103/PhysRevLett.78.5014

[2] Okada Y, Ekins-Daukes NJ, Kita T, et al. Intermediate band solar cells: recent progress and future directions. Appl Phys Rev. 2015;2(2):021302. doi: 10.1063/1.4916561

[3] Kita T, Harada Y, Asahi S. Energy conversion efficiency of solar cells. Singapore: Springer Singapore; 2019. doi: 10.1007/978-981-13-9089-0

[4] Ramiro I, Martí A. Intermediate band solar cells: present and future. Prog Photovolt Res Appl. 2021;29(7):705–713. doi: 10.1002/pip.3351

[5] Kada T, Asahi S, Kaizu T, et al. Two-step photon absorption in InAs/GaAs quantum-dot superlattice solar cells. Phys Rev B. 2015;91(20):201303. doi: 10.1103/PhysRevB.91.201303

[6] Kada T, Asahi S, Kaizu T, et al. Efficient two-step photocarrier generation in bias-controlled InAs/GaAs quantum dot superlattice intermediate-band solar cells. Sci Rep. 2017;7(1):5865. doi: 10.1038/s41598-017-05494-8 PMC5517490 28724895

[7] Yoshida M, Ekins-Daukes NJ, Farrell DJ, et al. Photon ratchet intermediate band solar cells. Appl Phys Lett. 2012;100(26):263902. doi: 10.1063/1.4731277

[8] Sogabe T, Hung CY, Tamaki R, et al. Experimental demonstration of energy-transfer ratchet intermediate-band solar cell. Commun Phys. 2021;4(1):38. doi: 10.1038/s42005-021-00543-1

[9] Fujiwara Y, Ito T, Ichida M, et al. Relaxation of optically excited *4f* electrons in Er-doped Ga*_x_*In_1−*x*_P. Phys B: Condens Matter. 1999;272(1–4):428–430. doi: 10.1016/S0921-4526(99)00360-9

[10] Ca NX, Hien NT, Fan X, et al. New insights on the luminescence properties and Judd–Ofelt analysis of Er-doped ZnO semiconductor quantum dots. RSC Adv. 2023;13(39):27292–27302. doi: 10.1039/D3RA05005J PMC10496136 37705985

[11] De Wit JW, Sonneveld LL, Meijerink A. Shedding light on host-to-Yb^3+^ energy transfer in Cs_2_AgBiBr_6_:Yb^3+^ (nano)crystals. Chem Mater. 2024;36(6):2857–2866. doi: 10.1021/acs.chemmater.3c03201 PMC10976640 38558916

[12] Jain A, Ong SP, Hautier G, et al. Commentary: the materials project: a materials genome approach to accelerating materials innovation. APL Mater. 2013;1(1):011002. doi: 10.1063/1.4812323

[13] Horton MK, Huck P, Yang RX, et al. Accelerated data-driven materials science with the materials project. Nat Mater. 2025;24(10):1522–1532. doi: 10.1038/s41563-025-02272-0 40610673

[14] Fujiwara Y. Room-temperature operation of injection-type 1.5 µm light-emitting diodes with Er, O-Codoped GaAs. Mater Trans. 2005;46(9):1969–1974. doi: 10.2320/matertrans.46.1969

[15] Takahei K, Taguchi A. Selective formation of an efficient Er-O luminescence center in GaAs by metalorganic chemical vapor deposition under an atmosphere containing oxygen. J Appl Phys. 1993;74(3):1979–1982. doi: 10.1063/1.354757

[16] Takahei K, Taguchi A, Horikoshi Y, et al. Atomic configuration of the Er-O luminescence center in Er-doped GaAs with oxygen codoping. J Appl Phys. 1994;76(7):4332–4339. doi: 10.1063/1.357319

[17] Morinaga Y, Edahiro T, Fujimura N, et al. Magnetic properties of Er and Er, O-doped GaAs grown by organometallic vapor phase epitaxy. Phys E Low-Dimens Syst Nanostructures. 2001;10(1):391–394. doi: 10.1016/S1386-9477(01)00123-0

[18] Smidstrup S, Markussen T, Vancraeyveld P, et al. QuantumATK: an integrated platform of electronic and atomic-scale modelling tools. J Phys Condens Matter. 2020;32(1):015901. doi: 10.1088/1361-648X/ab4007 31470430

[19] Synopsys Inc., Synopsys QuantumATK. Available from: https://www.synopsys.com/quantumatk

[20] Petukhov AG, Lambrecht WRL, Segall B. Electronic structure of rare-earth pnictides. Phys Rev B. 1996;53(8):4324–4339. doi: 10.1103/PhysRevB.53.4324 9983985

[21] Khalid S, Sharan A, Janotti A. Hybrid functional calculations of electronic structure and carrier densities in rare-earth monopnictides. Phys Rev B. 2020;101(12):125105. doi: 10.1103/PhysRevB.101.125105

[22] Eliseev PG, Gastev SV, Koizumi A, et al. Stimulated emission from GaAs:Er, O at 1538 nm. Quantum Electron. 2001;31(11):962–964. doi: 10.1070/QE2001v031n11ABEH002083

[23] Ralston JD, Ennen H, Wennekers P, et al. Structural, electrical and optical characterization of singlecrystal ErAs layers grown on GaAs by MBE. J Electron Mater. 1990;19(6):555–560. doi: 10.1007/BF02651278

[24] Ho VX, Dao TV, Jiang HX, et al. Photoluminescence quantum efficiency of Er optical centers in GaN epilayers. Sci Rep. 2017;7(1):39997. doi: 10.1038/srep39997 PMC5215594 28054672

[25] Culp TD, Cederberg JG, Bieg B, et al. Photoluminescence and free carrier interactions in erbium-doped GaAs. J Appl Phys. 1998;83(9):4918–4927. doi: 10.1063/1.367293

[26] Ofuchi H, Kubo T, Tabuchi M, et al. Local structures around Er atoms in GaAs:Er,O studied by fluorescence EXAFS and photoluminescence. Microelectron Eng. 2000;51–52:715–721. doi: 10.1016/S0167-9317(99)00535-3

[27] Iwahashi S, Kishi N, Kitayama S, et al. Multiple excitation process in deep-ultraviolet emission from AlGdN thin films pumped by an electron beam. J Appl Phys. 2012;111(8):083526. doi: 10.1063/1.4705416

[28] Gruber JB, Vetter U, Taniguchi T, et al. Spectroscopic analysis of Eu^3+^ in single-crystal hexagonal phase AlN. J Appl Phys. 2011;110(2):023104. doi: 10.1063/1.3609076

[29] Koizumi A, Fujiwara Y, Urakami A, et al. Room-temperature electroluminescence properties of Er,Ocodoped GaAs injection-type light-emitting diodes grown by organometallic vapor phase epitaxy. Appl Phys Lett. 2003;83(22):4521–4523. doi: 10.1063/1.1630165

[30] Boruc Z, Gawlik G, Fetliński B, et al. Temperature dependence of Er^3+^ ionoluminescence and photoluminescence in Gd_2_O_3_:Bi nanopowder. Rev Sci Instrum. 2014;85(6):064901. doi: 10.1063/1.4880456 24985836

[31] Takahei K, Taguchi A, Nakagome H, et al. Intra-4*f*-shell luminescence excitation and quenching mechanism of Yb in InP. J Appl Phys. 1989;66(10):4941–4945. doi: 10.1063/1.343765

[32] Klein PB. Time resolved photoluminescence from Yb^3+^ centers in InP:Yb Solid State Commun. 1988;65(10):1097–1101. doi: 10.1016/0038-1098(88)90901-5

[33] Aspnes DE, Studna AA. Dielectric functions and optical parameters of Si, Ge, GaP, GaAs, GaSb, InP, InAs, and InSb from 1.5 to 6.0 eV. Phys Rev B. 1983;27(2):985–1009. doi: 10.1103/PhysRevB.27.985

[34] Asahi S, Teranishi H, Kusaki K, et al. Two-step photon up-conversion solar cells. Nat Commun. 2017;8(1):14962. doi: 10.1038/ncomms14962 PMC5384214 28382945

[35] Fujiwara Y, Takemoto S, Nakamura K, et al. Ultrafast carrier trapping in Er-doped and Er,O-codoped GaAs revealed by pump and probe technique. Phys B. 2007;401–402:234. doi: 10.1016/j.physb.2007.08.155

[36] Radžiūtė L, Gaigalas G, Kato D, et al. Energy level structure of Er^3+^. J Quant Spectrosc Radiat Transf. 2015;152:94–106. doi: 10.1016/j.jqsrt.2014.11.007

[37] Taguchi A, Takahei K. Trap level characteristics of rare-earth luminescence centers in III–V semiconductors. J Appl Phys. 1996;79(8):4330–4334. doi: 10.1063/1.361741

